# Development and validation of the AF score for diagnosis of adult-onset Still's disease in fever of unknown origin^☆^

**DOI:** 10.1016/j.jtauto.2022.100184

**Published:** 2022-12-22

**Authors:** Shuni Ying, Duo Lv, Dingxian Zhu, Sheng Li, Yuwei Ding, Chuanyin Sun, Yu Shi, Hong Fang, Jianjun Qiao

**Affiliations:** a Department of Dermatology, The First Affiliated Hospital, Zhejiang University School of Medicine, Hangzhou, China; b Department of Clinical Pharmacy, The First Affiliated Hospital, Zhejiang University School of Medicine, Hangzhou, China; c Department of Dermatology, Shaoxing People's Hospital (Shaoxing Hospital, Zhejiang University School of Medicine), Shaoxing, China; d Department of Rheumatology, The First Affiliated Hospital, Zhejiang University School of Medicine, Hangzhou, China; e Department of Infectious Diseases, The First Affiliated Hospital, Zhejiang University School of Medicine, Hangzhou, China

**Keywords:** Adult-onset Still's disease, Fever of unknown origin, Persistent pruritic eruptions, Classification criteria, Bayesian method, AF score, AOSD/FUO score, AOSD, adult-onset Still's disease, BMA, Bayesian Model Averaging, CRP, C-reactive protein, ESR, erythrocyte sedimentation rate, FUO, fever of unknown origin, NPV, negative predictive value, PIP, posterior inclusion probability, PPEs, persistent pruritic eruptions, PPV, positive predictive value

## Abstract

**Objective:**

To develop and validate a diagnostic score to identify adult-onset Still's disease (AOSD) in fever of unknown origin (FUO).

**Methods:**

A single center, retrospective case-control study of inpatients with FUO from January 2018 to December 2021. Using clinical and laboratory data from 178 cases with AOSD and 486 cases with FUO, we developed an AOSD/FUO (AF) score with a Bayesian Model Averaging approach. AF score and Yamaguchi's criteria were evaluated by sensitivity, specificity, accuracy, and positive/negative predictive value for diagnosis of AOSD in developmental and validation samples.

**Results:**

Persistent pruritic eruptions (PPEs) in patients with rashes was higher in AOSD group than FUO group (52.3% vs 7.4%; *P* < 0.01). PPEs yielded a specificity of 97.5% and a sensitivity of 44.9%. AF score = PPEs × 3.795+Evanescent rash × 2.774+Serum ferritin × 1.678+Myalgia × 0.958+Neutrophil count × 0.185+Platelet count × 0.004. A cut-off value ≥ 5.245 revealed the maximizing sensitivity of 88.7% and specificity of 95.8% in discriminating AOSD from FUO in the validation group. And AF score improved the accuracy from 82.6% to 93.3% compared with Yamaguchi's criteria.

**Conclusions:**

We developed and validated a new score which can identify AOSD in FUO with higher classification accuracy than Yamaguchi's criteria. Future multi-centric prospective studies need to be designed to confirm the diagnosis value of AF score.

## Highlights

• Persistent pruritic eruptions is a clinical feature of adult-onset Still’s disease (AOSD).
• A weighted diagnostic score comprised 6 variables with fever as a prerequisite: persistent pruritic eruptions, evanescent rash, myalgia, neutrophil count, platelet count and serum ferritin. Score ≥5.245 is more likely to distinguish AOSD from fever of unknown.
• The accuracy of the weighted score of AOSD was improved from 82.6% to 93.3% comparing with Yamaguchi's criteria.

## 1 Introduction

Adult-onset Still's disease (AOSD) is a multisystem autoinflammatory disorder of unknown etiology [1]. The cardinal features including high spiking fever, joint symptoms, typical rash, sore throat, neutrophilic leukocytosis, and hyperferritinemia [2,3]. However, those features are not disease-specific enough to distinguish AOSD in the diseases with similar symptoms like infectious diseases (especially Epstein-Barr virus), rheumatic diseases, and Hodgkin lymphoma. How to diagnose AOSD quickly and accurately is still a difficult problem.

Fever of unknown origin (FUO) is a complex syndrome. It remains a diagnostic challenge especially patients with episodic fevers that “mimic” AOSD [[4], [5], [6]]. As for those diseases misdiagnosed as AOSD, especially the infectious diseases and hematological malignancy, patients may receive some steroids or immunosuppressive therapy, which can worsen the course. Due to the untimely diagnosis and treatment of AOSD, patients may suffer some severe complications with high mortality rate like macrophage activation syndrome, disseminated intravascular coagulopathy, and diffuse alveolar haemorrhage [3].

Several diagnostic criteria have been proposed with some noteworthy limitations in clinical testing. The most wildly used and best validated of proposed classification criteria is the Yamaguchi's criteria, a diagnosis of AOSD can be established after excluding other diseases [7,8], it may consume a lot of time and result in worse prognosis. Fautrel's criteria combines the evaluation of glycosylated ferritin fraction, which presents with a selection bias and isn't available in most centers [9]. A clinical scale was constructed to help distinguish patients with AOSD from FUO [10]. But its high specificity (98%) and low sensitivity (55%) [11] seems not suitable for screening patients.

Up to 87% of AOSD patients show with typical evanescent rash defined as a salmon-pink, macular or maculopapular eruption, tend to accompany with fever [7,12]. Recently, our cohort study and other reports demonstrated that an atypical type of rash occurred in AOSD [13,14]. The atypical persistent pruritic eruptions (PPEs) were dark-red flagellate macules, papules and/or plaques accompanied by itching, and persisting over 24 h without keeping step with fever. In histopathology, it is characterized by dyskeratotic keratinocytes in the upper one-third of the epidermis [[14], [15], [16]]. *Its diagnostic utility hasn't been described in any available classification criteria, and clinicians' insufficient awareness may cause a delay to differentiate patients with AOSD from those with FUO* [5].

In this study, we retrospectively analyzed 178 patients with AOSD and 486 patients with FUO without evidence of AOSD in our center over a 4-year period. We developed and validated a weighted classification score for AOSD.

## 2 Patients and methods

### 2.1 Patients

A single-center, retrospective review of 8182 inpatients treated for prolonged unexplained febrile illness from 2018 through 2021 was conducted. To be included in the study, patients should satisfy the criteria for confirming FUO [17], or the patient couldn't get an established diagnosis after one week of admission. Exclusion criteria were (1) younger than 18 years old or pregnant, (2) diagnosed with an established diagnosis before, (3) had started therapy with corticosteroid or immunosuppressive agents, and (4) human immunodeficiency virus positive.

AOSD was diagnosed according to the clinical features, the laboratory assessment, the effect of treatments, the disease courses, and complications by more than three experienced physicians basing on Yamaguchi's criteria, as the diagnostic gold standard. Patient with unclear diagnosis before discharge was given follow-up more than 6 months until a definitive diagnosis was established. All patients for whom a diagnostic doubt persisted were excluded. Patients were divided into “certain AOSD” group and those patients for whom an AOSD diagnosis was completely excluded were assigned to FUO group.

### 2.2 Patient characteristics

Clinical and laboratory parameters were extracted at the time of hospital admission. All the tests were performed in our center. Skin lesions were classified by two experienced dermatologists (SY and JQ). A patient was classified into PPEs group if he/she had special clinical manifestations like PPEs with (1) pathological manifestations as mentioned above, or (2) atypical/without pathological manifestations as mentioned above, after the exclusion of systemic lupus erythematosus, dermatomyositis and drug eruptions.

Those variables were evaluated for sensitivity, specificity, positive/negative predictive value (PPV, NPV), positive/negative likelihood ratio (PLR, NLR) according to the diagnostic gold standard.

### 2.3 Development and validation of the AOSD/FUO (AF) score

Bayesian Model Averaging approach (BMA) is a model construction method, which can assess the role of each variable and take the problem of model uncertainty into consideration, and then combines multiple model outputs into a new model to obtain a combined prediction model [18]. Posterior inclusion probabilities (PIP) represent probabilities of the variables should be included in all possible models.

70% patients were chosen randomly as developmental group, and the remaining patients were validation group. Due to the small sample size, we selected variables with statistically difference as parameters to use BMA. Using a ‘Monte Carlo Markov Chain’ method, we selected parameters with PIP greater than 0.7 to include as many valuable variables as possible. We got the coefficients of selected variables in the final AOSD/FUO (AF) score with binary logistic regression analysis. Cutoff point basing on maximizing sensitivity and specificity for discriminating AOSD from FUO was calculated using ROC curve analysis.

To validate the diagnostic value of AF score and Yamaguchi's criteria, we set PPEs and typical evanescent rash as the AOSD skin lesions as a primary diagnosis criterion on the basis of the original Yamaguchi's criteria. And we compared the statistical performance of AF score, Yamaguchi's criteria and modified Yamaguchi's criteria in the validation group.

### 2.4 Ethics

This study was performed according to the Declaration of Helsinki and local regulations. The study protocol was approved by the Ethics Committee of the First Affiliated Hospital, Zhejiang University School of Medicine (IIT-2021-272) according to the Medical Research Involving Human Subjects Act. All patients gave their written informed consent.

### 2.5 Statistics

Categorical variables were compared by Pearson chi-squared test or Fisher's test. Continuous variables were compared by Mann-Whitney *U* test or independent *t*-test. A p-value <0.05 was considered as statistically significant. We used IBM SPSS V.26.0 and R V.4.0.3 (with the R package ‘BMS’) for analyses.

## 3 Results

### 3.1 Population characteristics

**Fig. 1:**
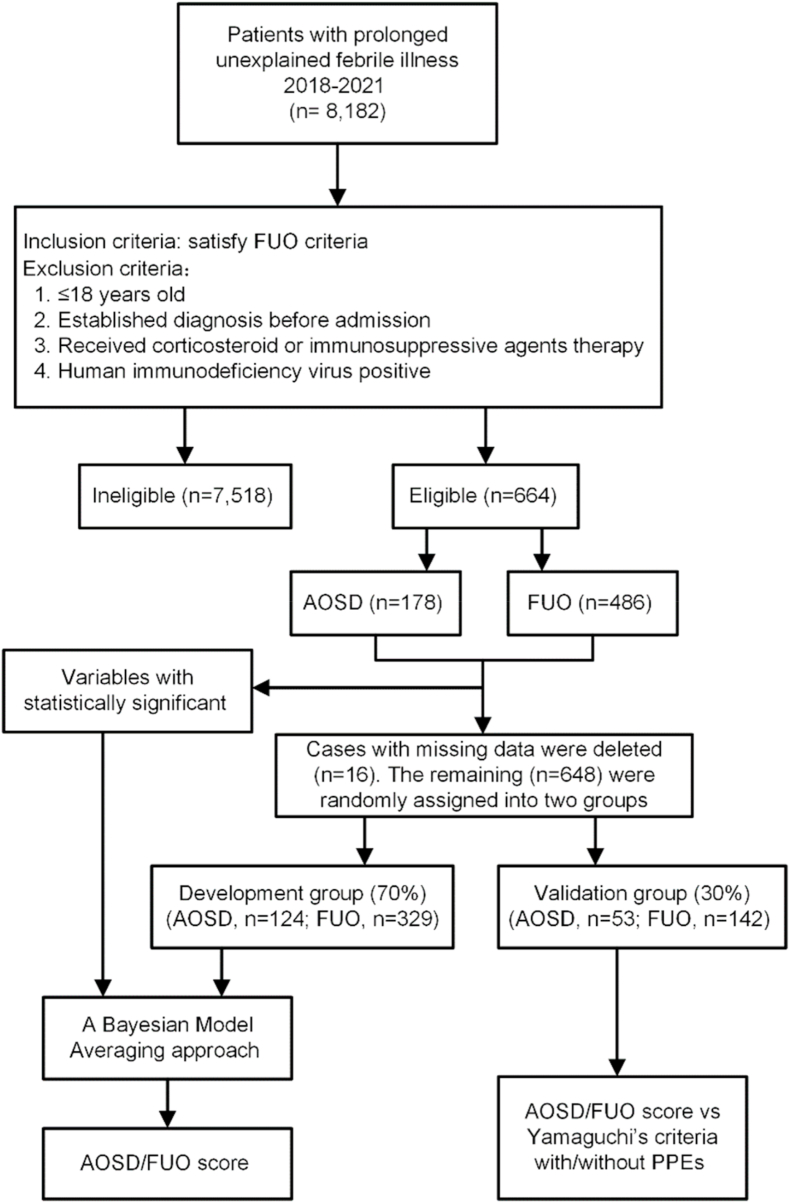
Flowchart of the development and validation of the AOSD/FUO (AF) score. Criteria for confirming FUO: a patient with temperature higher than 101.8 F (38.3 °C) for more than 3 weeks and no established diagnosis after one week of inpatient investigation.

A total of 664 patients (178 AOSD and 486 FUO) were recruited after informed consent was obtained (Fig. 1). They were listed in Supplementary Table 1.

### 3.2 Clinical and laboratory features

Clinical features and laboratory abnormalities were summarized in Table 1. AOSD was more common in young women with higher temperature and more hospitalization days (*P* < 0.01). The symptoms of sore throat, myalgia, arthritis/arthralgias, lymphadenopathy, and rash were more common in AOSD (*P* < 0.01). The percentage of PPEs was higher than evanescent rash in AOSD, and the percentage of PPEs in patients with rashes in AOSD group was higher than those in FUO group (*P* < 0.01). No statistically difference of frequency was found between the two groups in hepatomegaly, pneumonitis, pericarditis, stomachache or malignancies.

**Table 1:** Clinical features and laboratory tests of patients with AOSD and with FUO.

Variables	AOSD (n = 178)	FUO (n = 486)	*P* value
Females	124 (69.7)	268 (55.1)	0.001
Age (mean ± SD, years)	43.1 ± 16.1	49.4 ± 18.0	<0.001
Fever (mean ± SD, °C)	39.6 ± 0.7	39.3 ± 0.7	<0.001
Fever days before hospital (mean ± SD, days)	29.3 ± 58.0	21.7 ± 27.7	0.463
Fever days in hospital (mean ± SD, days)	9.0 ± 6.3	6.6 ± 5.0	<0.001
Hospitalization days (mean ± SD, days)	15.9 ± 7.0	12.2 ± 6.3	<0.001
Sore throat	75 (42.1)	76 (15.6)	<0.001
Myalgia	87 (48.9)	105 (21.6)	<0.001
Arthritis or arthralgias	88 (49.4)	86 (17.7)	<0.001
Lymphadenopathy	143 (80.3)	305 (62.8)	<0.001
Splenomegaly	113 (63.5)	259 (53.3)	0.019
Hepatomegaly	11 (6.2)	38 (7.8)	0.584
Rash	153 (86.0)	162 (33.3)	<0.001
Evanescent rash	56/178 (31.5)	31/486 (6.4)	<0.001
Persistent pruritic eruption	80/178 (44.9)	12/486 (2.5)	<0.001
Other rashes	17/178 (9.6)	119/486 (24.5)	<0.001
Pneumonitis	59 (33.1)	174 (35.8)	0.525
Pericarditis	27 (15.2)	76 (15.6)	0.978
Stomachache	2 (1.1)	15 (3.1)	0.265
Tumor	3 (1.7)	5 (1.0)	0.448
Leukocytes, × 10^9^/L	13.65 (10.08–17.60)	7.10 (4.37–10.10)	<0.001
Neutrophil count, × 10^9^/L	11.75 (7.90–15.62)	4.50 (2.60–7.30)	<0.001
Neutrophil count percent, %	85.45 (79.45–90.00)	72.55 (59.00–80.50)	<0.001
Haemoglobin, g/dL	110.43 ± 18.55	114.0 ± 20.56	0.042
Red blood cell count, × 10^12^/L	3.88 ± 0.56	3.92 ± 0.68	0.498
Platelet count, × 10^9^/L	274.50 (203.75–366.25)	211.00 (128.00–290.50)	<0.001
CRP, mg/L	85.45 (47.83–139.00)	57.60 (21.38–109.85)	<0.001
ESR, mm/hour	65.50 (37.50–87.25)	37.00 (14.75–77.25)	<0.001
Serum ferritin, μg/L	4955.15 (2156.03–14108.93)	675.15 (315.20–1991.58)	<0.001
Fibrinogen, mg/L	5.18 (4.14–6.51)	4.31 (3.02–5.79)	<0.001
D-dimer, ng/mL	2851.00 (1562.50–7150.75)	1571.00 (840.75–3362.50)	0.001
ALT, units/L	37.00 (20.75–70.75)	33.00 (16.75–67.00)	0.107
AST, units/L	40.50 (26.00–69.00)	30.00 (19.00–61.00)	0.001
Triglycerides	1.26 (0.93–1.78)	1.23 (0.91–1.73)	0.886
CREA	55.00 (47.00–65.00)	63.00 (52.00–77.00)	<0.001
LDH, units/L	420.00 (300.50–615.00)	288.00 (206.00–455.00)	<0.001
CK	29.00 (21.00–45.00)	40.00 (26.00–64.00)	<0.001
CK-MB	15.00 (11.00–20.00)	14.00 (10.00–19.00)	0.006
Hydroxybutyrate dehydrogenase	316.00 (223.00–463.00)	235.00 (165.00–367.00)	<0.001
PCT	0.20 (0.10–0.54)	0.16 (0.07–0.33)	0.001

*Data of categorical variables are expressed as number positive/number with information available (%) unless otherwise specified. Data of continuous variables are expressed as medians (interquartile ranges).

Categorical variables were compared by Pearson chi-squared test or Fisher's test. Continuous variables were compared by Mann-Whitney *U* test or independent *t*-test (for variables conformed to the normal distribution).

CRP, C-reactive protein; ESR, erythrocyte sedimentation rate; ALT, alanine aminotransferase; AST, aspartate aminotransferase; CREA, creatinine; LDH, lactate dehydrogenase; CK, creatine kinase; CK-MB, creatine kinase isoenzyme-MB; PCT, procalcitonin; SD, standard deviation.

Laboratory findings showed that inflammatory biomarkers, including leukocyte count, neutrophil count, CRP, ESR, serum ferritin levels, lactate dehydrogenase (LDH) and procalcitonin (PCT), and other variables including platelet count, aspartate aminotransferase (AST), fibrinogen, and D-dimer were higher in patients with AOSD (*P* < 0.01). Creatinine and creatine kinase were found higher in patients with FUO (*P* < 0.01).

### 3.3 Diagnostic efficacy of clinical and laboratory parameters

Clinical features and laboratory parameters that were sensitive for establishing diagnosis of AOSD included high level of serum ferritin≥ the upper normal limit (97.7%), CRP≥ the upper normal limit (96%), D-dimer≥ the upper normal limit (92.1%), ESR≥ the upper normal limit (91.6%), and hyperpyrexia (≥39 °C, 90.4%) (Supplementary Table 2). Parameters with the highest specificity was PPEs (97.5%), followed with sore throat (84.4%), pericarditis (84.4%). The highest PPV (87%) and PLR (17.96) was for PPEs. Serum ferritin≥ the upper normal limit showed with the highest NPV (97.7%) and the lowest NLR (0.08).

### 3.4 Development of the AF score

The BMA method requires complete data for calculation, 16 patients (1 AOSD and 15 FUO) with missing data were excluded. The developmental dataset was composed of 124 AOSD and 329 FUO, and the remaining 30% of patients (53 AOSD and 142 FUO) were validation dataset. 31 variables with statistically difference were selected and more than 21 billion (2^31^) models were computed, and PIP of the variables in all possible models were presented in *Supplementary* Table 3.

Since the value of serum ferritin level varies widely (ranging from 0 to 40000 ng/ml in our hospital), and the best cutoff point is still under debate, we developed 5 diagnostic models containing variable of different ferritin value: above the upper limit of normal, higher than 1000 ng/ml, higher than 1500 ng/ml, higher than 2000 ng/ml, and higher than 2500 ng/ml. Their diagnostic efficacy in the development group and in the validation group were showed in Table 2*.* We found model 4 yielded the highest specificity both in the development group (91.2%) and validation group (95.8%), and the highest accuracy in the validation group (93.3%).

**Table 2:** Comparison the diagnostic efficacy of AOSD with different models in the development and validation group.

	AUC	Cut-off point	Sensitivity		Specificity		Accuracy	
			DG	VG	DG	VG	DG	VG
Model 1	0.947	4.793	0.887	0.906	0.878	0.908	0.881	0.908
Model 2	0.982	5.250	0.944	0.962	0.906	0.852	0.916	0.882
Model 3	0.983	5.971	0.968	0.811	0.894	0.957	0.914	0.918
Model 4	0.955	5.245	0.895	0.887	0.912	0.958	0.907	0.933
Model 5	0.954	5.229	0.895	0.868	0.909	0.958	0.905	0.933

Model 1: serum ferritin as continuous variable; Model 2: serum ferritin ≥1000 ng/ml; Model 3: serum ferritin≥ 1500 ng/ml; Model 4: serum ferritin≥ 2000 ng/ml; Model 5: serum ferritin≥ 2500 ng/ml.

* With only evanescent rash as a major criterion of Yamaguchi's criteria.

** Skin lesions include evanescent rash and PPEs, it is considered to meet the criterion of Still's rash if one of them occurs.

AUC, area under curve; DG, development group; VG, validation group.

**Table 3:** Logistic regression analysis with the best combination of clinical and laboratory variables for developing the weighted criteria of AOSD.

	β-coefficient	*p*	Odds Ratio	95% CI
Persistent pruritic eruption	3.795	<0.001	44.467	16.867–117.232
Evanescent rash	2.774	<0.001	16.027	6.823–37.645
Serum ferritin≥ 2000 ng/ml	1.678	<0.001	5.355	2.523–11.367
Myalgia	0.958	0.009	2.607	1.272–5.344
Neutrophil count	0.185	<0.001	1.203	1.116–1.297
Platelet count	0.004	0.015	1.004	1.001–1.008

All variable were included in model analysis.

We defined model 4 as final AF score. The β coefficients and the 95%CI of all the variables in AF score were shown in Table 3. AF score showed with fever as a prerequisite as following: AFscore=PPEs×3.795+Evanescentrash×2.774+Serumferritin×1.678+Myalgia×0.958+Neutrophilcount×0.185+Plateletcount×0.004. A score of 1 or 0 is depending on whether the clinical variable is present or absent (serum ferritin's score is 1 when the level is higher than 2000 ng/ml). For platelet count and neutrophil count, the values with unit mentioned in Table 1. The area under the curve of the model was 0.955 (95%CI = 0.937–0.973) in the developmental sample. The cut-off was 5.245, with the maximizing sensitivity and specificity (Table 4).

**Table 4:** Proposed new set of criteria for AOSD classification.

Variables	Value	Coefficient
Persistent pruritic eruption	0/1	3.795
Evanescent rash	0/1	2.774
Serum ferritin≥ 2000 ng/ml	0/1	1.678
Myalgia	0/1	0.958
Neutrophil count	observed values	0.185
Platelet count	observed values	0.004

Calculation of the AF score = Persistent pruritic eruption × 3.795+Evanescent rash × 2.774+Serum ferritin × 1.678+Myalgia × 0.958+Neutrophil count × 0.185+Platelet count × 0.004, with fever as a prerequisite. In the formula, 1 or 0 is placed for binary variables and observed values for continuous variables. Cut-off point is 5.245.

### 3.5 AF score versus Yamaguchi criteria with/without PPEs in the development and validation group

The percentage of PPEs in AOSD patients with rashes was higher than patients with FUO both in development group (43.5% versus 3.3%; p < 0.01) and validation cohort (49.1% versus 0.7%; p < 0.01). Their specificity (96.7% versus 99.3%) in two groups showed similarly. The modified Yamaguchi's criteria showed with higher sensitivity, NPV, and accuracy than classical Yamaguchi (Table 5).

**Table 5:** AF score performed better than Yamaguchi's criteria in the development and validation setting.

	Sensitivity		Specificity		PPV		NPV		Accuracy	
	DG	VG	DG	VG	DG	VG	DG	VG	DG	VG
AF score	0.895	0.887	0.912	0.958	0.793	0.870	0.958	0.957	0.907	0.933
Yamaguchi^a^	0.831	0.906	0.781	0.796	0.589	0.623	0.924	0.958	0.795	0.826
Modified Yamaguchi^b^	0.903	0.943	0.763	0.796	0.589	0.633	0.954	0.974	0.801	0.836

PPV/NPV, positive/negative predictive value.

DG, development group; VG, validation group.

a With only evanescent rash as a major criterion of Yamaguchi's criteria.

b Skin lesions include evanescent rash and PPEs, it is considered to meet the criterion of Still's rash if one of them occurs.

As defined, AF score showed with a sensitivity of 89.5%, a specificity of 91.2%, PPV and NPV of 79.3% and 95.8%, and accuracy of 90.7% in the development group. These values are higher than those for clinical Yamaguchi's criteria in the same population, which displayed similarly in the validation group. On the basis of Yamaguchi's set, AF score established AOSD diagnosis for another 22 false negative cases, which improved the accuracy from 82.6% to 93.3% in the validation group. The comparison of AF score with Yamaguchi's criteria and modified Yamaguchi's criteria is shown with ROC curves in Supplementary Fig. 1.

## 4 Discussion

In this study, we compared the clinical, and laboratory characteristics of patients with AOSD of the patients with FUO in the same period, and developed a specific classification score for diagnosing AOSD. The performance of the AF score was confirmed better than Yamaguchi's criteria both in the development group and validation group.

Almost all the patients with AOSD suffer high spiking fever during disease courses (93%–100%) [3], and they can account up to 20% of FUO [5,19,20]. Although fever was not selected to compose the final score in our study, it was still an essential prerequisite for using AF score.

Recent reports showed that the appearance of PPEs often associates with worse prognosis in patients with AOSD [14,21]. PPEs showed with highest specificity and PPV when compared the diagnostic utility with that of other AOSD characteristics, which indicated that PPEs was very predictive in diagnosis. However, PPEs was not yet incorporated in any classification criteria of AOSD [3,7,12]. So, we added PPEs in the type of Still's rash basing on Yamaguchi's criteria, which increased sensitivity and accuracy than before. It is proved that PPEs has a certain prompt value for the diagnosis of AOSD.

Hyperferritinemia is considered to be one of the important diagnostic markers for AOSD [3,22]. The patient is more likely to be considered to have AOSD when the serum ferritin exceeds 5 times the normal value [23]. Hyperferritinemia may be the result of inflammatory response, or it may exacerbate the inflammatory response by promoting a cytokine storm [23]. Serum ferritin levels may correlate with disease activity and severity [1,22]. In our study, we analyzed the diagnostic efficacy in different models with different cutoff points of serum ferritin. And we found that ferritin level ≥2000 ng/ml appeared with better prediction for a diagnosis of AOSD.

Several features including arthritis or arthralgias, sore throat and splenomegaly were not set as criteria in AF score. A deserving point to emphasize is that FUO is a complex syndrome with myriad etiologies [8]. Though some symptoms are special in some diseases, they may lose statistically significance after mixing with other cases in FUO group when compared with AOSD group.

The limitations of this study include single‐center retrospective study with a small sample size to generate and test the score, and many control cases remained with unknown diagnosis due to insufficient follow-up time. Future prospective studies need to be designed to clarify its sensitivity, specificity, and predictive value.

## 5 Conclusion

In conclusion, our study show that PPEs seems a useful marker of AOSD and the AF score is a numeric, simple and objective tool that may help identify a diagnosis of AOSD in patients with FUO better than Yamaguchi's criteria. The validation in different populations is required to test the capacity of AF score to obtain widespread acceptance and use in clinical research. However, due to the limited number of patients and the single‐center design, future multi-centric prospective studies are required to validate AF score in different populations to obtain widespread acceptance and use in clinical research.

## Credit author statement

Shuni Ying: Supervision, Analysis and interpretation of data, Writing- Reviewing and Editing; Duo Lv: Writing - Original Draft, Analysis and interpretation of data; Dingxian Zhu: Acquisition of data; Sheng Li: Acquisition of data; Yuwei Ding: Acquisition of data; Chuanyin Sun: Writing - Original Draft; Yu Shi: Writing - Original Draft; Hong Fang: Writing- Reviewing and Editing; Jianjun Qiao: Supervision.

## Funding sources

This work was supported by 10.13039/501100001809National Natural Science Foundation of China (82173400 to JQ, 81972931 to HF), the 10.13039/501100014996Medical and Health Science and Technology Project of Health Commission of Zhejiang Province (2020KY558 to JQ).

## Declaration of compering interest

The authors declare that they have no known competing financial interests or personal relationships that could have appeared to influence the work reported in this paper.

## Data Availability

Data will be made available on request.
