# Supplementary material for: Development and validation of the AF score for diagnosis of adult-onset Still's disease in fever of unknown origin
Source: J Transl Autoimmun. 2022 Dec 22;6:100184. doi: 10.1016/j.jtauto.2022.100184 (PMC9826851; doi:10.1016/j.jtauto.2022.100184)
Supplement: Multimedia component 1 [file mmc1.docx]

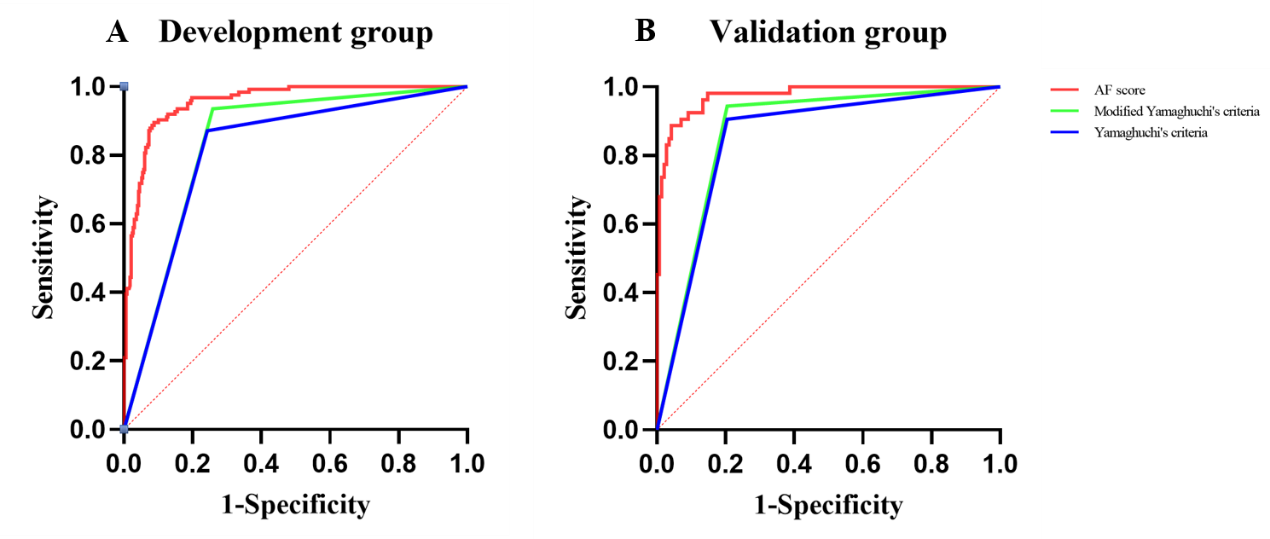


Supplementary figure 1. AF score performed better when compared with Yamaguchi’s criteria in different groups. (A) Receiver operating characteristic (ROC) curves for the development group. Area under the curve: AF score, 0.955 (0.937-0.973); Yamaguchi’s criteria, 0.814 (0.770-0.858); modified Yamaguchi’s criteria, 0.839 (0.800-0.877). (B) In the development group, area under the curve: AF score, 0.975 (0.955-0.995); Yamaguchi’s criteria, 0.851 (0.790-0.911); modified Yamaguchi’s criteria, 0.870 (0.815-0.924).
