# Supplementary material for: Development and validation of the AF score for diagnosis of adult-onset Still's disease in fever of unknown origin
Source: J Transl Autoimmun. 2022 Dec 22;6:100184. doi: 10.1016/j.jtauto.2022.100184 (PMC9826851; doi:10.1016/j.jtauto.2022.100184)
Supplement: Multimedia component 2 [file mmc2.docx]

Supplementary table 1. Diagnosis of patients in the control group

| Classification of diseases in the control group | Diagnosis |
| --- | --- |
| Infections (n=227) | Epstein-Barr virus infections (n=31) |
|  | cytomegalovirus infections (n=15) |
|  | tuberculosis infection (n=10) |
|  | salmonella infections (n=5) |
|  | brucellosis (n=5) |
|  | rickettsiosis infections (n=3) |
|  | adenovirus infection (n=1) |
|  | cryptococcosis (n=1) |
|  | toxoplasmosis (n=1) |
|  | herpes simplex virus infection (n=1) |
|  | bacteremia with unknown bacteria (n=46) |
|  | viral infections with unknown origin (n=35) |
|  | infections with indefinite origins (n=73) |
| Systemic diseases (n=159) | vasculitis (n=17) |
|  | undifferentiated connective tissue diseases (n=16) |
|  | necrotizing lymphadenitis (n=14) |
|  | systemic lupus erythematosus (n=10) |
|  | polymyalgia rheumatica (n=10) |
|  | Sjögren syndrome (n=9) |
|  | dermatomyositis (n=6) |
|  | mixed connective tissue disease (n=6) |
|  | macrophage activation syndromes (n=4) |
|  | amyopathic dermatomyositis (n=2) |
|  | IgG4 related disease (n=2) |
|  | primary biliary cirrhosis (n=2) |
|  | Sweet syndrome (n=2) |
|  | polymyositis (n=1) |
|  | Reiter syndrome (n=2) |
|  | rheumatoid arthritis (n=1) |
|  | anti-synthetase syndrome (n=1) |
|  | Evans syndrome (n=1) |
|  | allergic arthritis (n=1) |
|  | ankylosing spondylitis (n=1) |
|  | nodular panniculitis (n=1) |
|  | systemic diseases with indefinite diagnosis (n=50) |
| Neoplasms (n=68) | Hodgkin lymphoma (n=37) |
|  | non-Hodgkin’s lymphoma (n=25) |
|  | Castleman’s disease (n=6) |
| Miscellaneous diagnosis (n=5) | drug eruption (n=2) |
|  | hypopituitarism (n=1) |
|  | drug hepatitis (n=1) |
|  | Monoclonal gammopathy (n=1) |
| Unexplained fever (n=27) | unexplained fevers (n=27) |
