# Supplementary material for: Development and validation of the AF score for diagnosis of adult-onset Still's disease in fever of unknown origin
Source: J Transl Autoimmun. 2022 Dec 22;6:100184. doi: 10.1016/j.jtauto.2022.100184 (PMC9826851; doi:10.1016/j.jtauto.2022.100184)
Supplement: Multimedia component 3 [file mmc3.docx]

Supplementary table 2. Analysis of variables on the diagnosis of AOSD

| Variable | AOSD  No. of Cases | FUO  No. of Cases | Sensitivity, % | Specificity, % | PPV, % | NPV, % | PLR | NLR | Youden index |
| --- | --- | --- | --- | --- | --- | --- | --- | --- | --- |
| Females | 124 | 268 | 69.7 | 44.9 | 31.6 | 80.1 | 1.26 | 0.67 | 0.146 |
| Fever≥39℃ | 161 | 368 | 90.4 | 24.3 | 30.4 | 87.4 | 1.19 | 0.40 | 0.147 |
| Sore throat | 75 | 76 | 42.1 | 84.4 | 49.7 | 79.9 | 2.70 | 0.69 | 0.265 |
| Myalgia | 87 | 105 | 48.9 | 78.4 | 45.3 | 80.7 | 2.26 | 0.65 | 0.273 |
| Arthritis or arthralgias | 88 | 86 | 49.4 | 82.3 | 50.6 | 81.6 | 2.79 | 0.61 | 0.317 |
| Lymphadenopathy | 143 | 305 | 80.3 | 37.2 | 31.9 | 83.8 | 1.28 | 0.53 | 0.175 |
| Splenomegaly or hepatomegaly | 113 | 264 | 63.5 | 45.7 | 30.0 | 77.4 | 1.17 | 0.80 | 0.092 |
| Evanescent rash | 56 | 31 | 31.5 | 80.9 | 64.4 | 78.9 | 1.65 | 0.85 | 0.124 |
| Persistent pruritic eruption | 80 | 12 | 44.9 | 97.5 | 87.0 | 82.9 | 17.96 | 0.57 | 0.424 |
| Pneumonitis | 59 | 174 | 33.1 | 64.2 | 25.3 | 72.4 | 0.92 | 1.04 | -0.027 |
| Pericarditis | 27 | 76 | 15.2 | 84.4 | 26.2 | 73.1 | 0.97 | 1.00 | -0.004 |
| Leukocytes＞10,000/mm^3^ | 135 | 125 | 75.8 | 74.3 | 51.9 | 89.4 | 2.95 | 0.33 | 0.501 |
| Neutrophil count percent＞80% | 132 | 132 | 74.2 | 72.8 | 50.0 | 88.5 | 2.73 | 0.35 | 0.47 |
| CRP＞N | 171 | 433 | 96.0 | 10.9 | 28.3 | 88.3 | 1.08 | 0.37 | 0.069 |
| ESR＞N | 163 | 355 | 91.6 | 26.3 | 31.5 | 89.7 | 1.24 | 0.32 | 0.179 |
| Fibrinogen＞N | 136 | 268 | 76.4 | 44.7 | 33.7 | 83.8 | 1.38 | 0.53 | 0.211 |
| D-dimer＞N | 164 | 395 | 92.1 | 17.9 | 29.3 | 86.7 | 1.12 | 0.44 | 0.1 |
| Serum ferritin＞N | 174 | 316 | 97.7 | 28.2 | 35.5 | 97.7 | 1.36 | 0.08 | 0.259 |
| Serum ferritin＞1000 ng/ml | 158 | 182 | 88.8 | 58.6 | 46.5 | 93.8 | 2.14 | 0.19 | 0.474 |
| Serum ferritin＞1500 ng/ml | 144 | 145 | 80.9 | 67.0 | 49.8 | 90.9 | 2.45 | 0.29 | 0.479 |
| Serum ferritin＞2000 ng/ml | 137 | 120 | 77.0 | 72.7 | 53.3 | 89.9 | 2.82 | 0.32 | 0.497 |
| Elevated liver enzymes | 109 | 237 | 61.2 | 51.2 | 31.5 | 78.3 | 1.25 | 0.76 | 0.124 |
| LDH＞N | 149 | 282 | 84.2 | 40.0 | 34.6 | 87.6 | 1.40 | 0.40 | 0.242 |
| PCT＞N | 125 | 80 | 71.4 | 82.7 | 61.0 | 88.5 | 4.13 | 0.35 | 0.541 |
| Negative ANA and RF | 159 | 398 | 89.3 | 15.7 | 28.5 | 82.2 | 1.06 | 0.68 | 0.069 |

PPV/NPV, positive/negative predictive value. PLR/NLR, positive/negative likelihood ratio.

Normal CRP value were 0-8 mg/L; Normal ESR value were 0-15 mm/h; Normal Fibrinogen value were 2-4 g/L; Normal D-dimer value were 0-700 μg/L (FEU); Normal serum ferritin value were 7-323 ng/ml; Normal ALT value were 9-40 U/L, normal AST value were 15-40 U/L; Normal LDH value were 120-250 U/L; Normal CK value were 50-310 U/L; Normal PCT value were 0-0.5 ng/ml.
