# Supplementary material for: Development and validation of the AF score for diagnosis of adult-onset Still's disease in fever of unknown origin
Source: J Transl Autoimmun. 2022 Dec 22;6:100184. doi: 10.1016/j.jtauto.2022.100184 (PMC9826851; doi:10.1016/j.jtauto.2022.100184)
Supplement: Multimedia component 4 [file mmc4.docx]

Supplementary table 3. Posterior probabilities of the demographic, clinical and laboratory variables obtained through the BMA

|  | Posterior probability |
| --- | --- |
| Demographic characteristics |  |
| Female | 0.032 |
| Age at onset | 0.219 |
| Clinical features |  |
| Sore throat | 0.363 |
| Myalgia | 0.736 |
| Arthritis or arthralgias | 0.453 |
| Lymphadenopathy | 0.234 |
| Splenomegaly | 0.580 |
| Evanescent rash | 1.000 |
| Persistent pruritic eruption | 1.000 |
| Other rashes | 0.040 |
| Fever | 0.474 |
| Fever days in hospital | 0.112 |
| Hospitalization days | 0.059 |
| Laboratory tests |  |
| White blood cell count | 0.175 |
| Neutrophil count | 0.915 |
| Neutrophil percent | 0.033 |
| Platelet count | 0.741 |
| Hemoglobin | 0.017 |
| AST | 0.328 |
| C-reactive protein | 0.018 |
| Erythrocyte sedimentation rate | 0.054 |
| Serum ferritin | 1.000 |
| Fibrinogen | 0.100 |
| D-dimer | 0.117 |
| Creatinine | 0.042 |
| Lactate dehydrogenase | 0.028 |
| Creatine kinase | 0.090 |
| Creatine kinase isoenzyme-MB | 0.070 |
| Hydroxybutyrate dehydrogenase | 0.024 |
| T-spot negative | 0.136 |
| PCT | 0.086 |

Posterior inclusion probabilities represent probabilities of the variables should be included in all possible models, we selected parameters whose PIP greater than 0.7 for regression analysis to include as many valuable variables as possible.
